# The correlation between lower limb spasticity and proprioceptive dysfunction in post-stroke patients

**DOI:** 10.3389/fneur.2025.1634382

**Published:** 2025-08-20

**Authors:** Shiai Gao, Zifu Yu, Xihua Liu

**Affiliations:** ^1^School of Rehabilitation Medicine, Shandong University of Traditional Chinese Medicine, Jinan, China; ^2^Shandong Provincial Center for Disease Control and Prevention, Jinan, China; ^3^Department of Rehabilitation, Affiliated Hospital of Shandong University of Traditional Chinese Medicine, Jinan, China

**Keywords:** stroke, spasms of the lower limbs, proprioception, musculoskeletal ultrasound, correlation analysis

## Abstract

**Objective:**

To investigate the correlation between lower limb spasticity and proprioception in stroke patients, to analyze the influencing factors of spasticity, and to evaluate the predictive value of musculoskeletal ultrasound parameters on spasticity.

**Methods:**

A cross-sectional study was used to enroll 80 stroke patients admitted to the Rehabilitation Center of the Affiliated Hospital of Shandong University of Traditional Chinese Medicine from October 2024 to April 2025. The degree of lower limb spasticity was evaluated by the modified Ashworth Scale (MAS), and the proprioceptive assessment module of the Pro-kin balance system was used to quantitatively detect the mean trajectory error (ATE) and the average weight-bearing asymmetry (AWA). Musculoskeletal ultrasound was used to detect the spasmodic side and the healthy gastrocnemius muscle, and the longitudinal and transverse ultrasound images were obtained, and the length of muscle fibers, medial head pinnate angle and muscle thickness were measured. Spearman correlation analysis was used to explore the correlation between MAS score and various parameters, a multiple linear regression model was constructed to analyze the influencing factors of spasticity, and the predictive performance of ultrasound parameters was evaluated by receiver operating characteristic (ROC) curve.

**Results:**

Compared with the healthy side, the muscle fiber length, medial head pinnate angle and muscle thickness of the gastrocnemius muscle on the spasticity side were significantly reduced (*p* < 0.05). The mean trajectory difference of proprioceptive parameters was (65.83 ± 13.11) %, and the average weight-bearing force difference was (2.41 ± 0.46) kg. Correlation analysis showed that MAS spasticity score was statistically significantly related to proprioceptive parameters and muscle structure parameters (*p* < 0.05), and multiple linear regression analysis showed that muscle feather angle (*β* = 0.362), muscle fiber length (*β* = −0.157), muscle thickness (*β* = −0.230), mean trajectory error (*β* = 0.329) and average weight-bearing strength difference (*β* = 0.260) constituted independent influencing factors for MAS score (adjusted *R^2^* = 0.787, *P*<0.05). ROC curve analysis showed that the area under the curve of muscle pinnate angle predicting spasticity (MAS ≥ 2 grade) was the largest (AUC = 0.850, 95%CI: 0.729–0.972), which was better than muscle fiber length (AUC = 0. 840) and muscle thickness (AUC = 0.838).

**Conclusion:**

There is a significant correlation between lower limb spasticity and proprioceptive and muscle structure parameters in stroke patients, and proprioception and muscle structure parameters are the key factors affecting spasticity, and musculoskeletal ultrasound can be used as a quantitative evaluation tool for lower limb spasticity in stroke.

**Clinical trial registration:**

https://itmctr.ccebtcm.org.cn/.

## 1 Introduction

Stroke has a high rate of disability, and the motor dysfunction caused by stroke seriously threatens the quality of life of patients (1). Studies have shown that about 70% of patients are left with lower extremity dysfunction, with spastic paralysis being a common complication, characterized by persistent hypertonia, limited range of motion, and abnormal postural control (2). Previous studies have noted that patients with post-stroke patients often have abnormalities in lower extremity proprioceptive function (3). The proprioceptive system plays an important role in regulating motor control by integrating proprioceptor information from muscles, tendons, and joints (4, 5). However, the current research on lower limb spasticity in stroke mostly focuses on the regulation of muscle tone, such as botulinum toxin injection, antispasmodic drugs, muscle stretching and physical factor therapy, etc., and the attention to proprioception is relatively insufficient, and the key role of sensory afferents in central nervous system remodeling is ignored, resulting in a lack of evidence-based evidence for the association mechanism between spasticity and proprioceptive disorders.

In recent years, biofeedback-based neurorehabilitation technology has provided a new approach for sensory-motor integration research. The Pro-Kin Balance System uses multi-axis pressure sensing technology combined with real-time visual feedback to objectively and quantitatively assess a patient’s postural control (6). Most of the existing studies have used the Pro-Kin system to assess static balance function, but the system can also assess the patient’s proprioception (7), compared with the scale evaluation, it can more accurately and objectively reflect the patient’s proprioceptive function. To this end, the Pro-Kin balance system was used to evaluate the proprioceptive function of the affected side of patients with spasticity.

In addition, the modified Ashworth Scale (MAS) has been widely used in clinical practice, and its reliability and validity are high, but it is difficult to accurately quantify the neuromuscular characteristics of spasticity due to subjective judgment (8). As an emerging evaluation method, musculoskeletal ultrasound can objectively reflect the changes in muscle structure and mechanical properties by measuring muscle thickness, pinnate angle, muscle fiber length and other parameters (9). Studies have shown that (10), There was a significant correlation between the medial head shear wave velocity of the gastrocnemius muscle and the degree of ankle plantar flexor spasm, suggesting that ultrasound parameters may be a biomarker for predicting the degree of spasticity, but its predictive performance and parameter selection criteria in lower limb spasticity after stroke still need to be further verified. In addition, in clinical practice, the heterogeneity of spasticity degree is influenced by multiple factors, and this study will verify whether proprioception and muscle structure measurement parameters are factors influencing spasticity degree.

Based on this, this study aimed to explore the intrinsic relationship between the degree of lower limb spasticity and proprioceptive impairment in stroke patients, systematically analyze the clinical factors affecting the degree of spasticity, and explore the predictive value of musculoskeletal ultrasound parameters on the degree of spasticity. The results of this study will provide a theoretical basis for the formulation of precise rehabilitation strategies, and open up a new path for the establishment of a spasticity assessment system based on objective indicators.

## 2 Information and methodology

### 2.1 Subjects of the study

From October 2024 to April 2025, 80 patients with lower limb spasm after stroke in the Rehabilitation Center of the Affiliated Hospital of Shandong University of Traditional Chinese Medicine were enrolled. Inclusion Criteria: (1) Comply with the “Key Points for the Diagnosis of Major Cerebrovascular Diseases in China 2019” (11) diagnostic criteria for intracerebral hemorrhage or cerebral infarction, all confirmed by cranial CT or MRI; (2) The first onset, the course of the disease ≤ 6 months, unilateral lower limb involvement; (3) Age 25 ~ 80 years old; (4) Patients with stable vital signs, a score of ≥24 on the Simplified Intelligent Mental State Examination (MMSE), and no serious heart, brain, and renal complications; (5) Able to sit or stand independently or with assistance; (6) Both the patient and his family are aware of and voluntarily participate in the study and sign the informed consent form. Exclusion Criteria: (1) Severe cardiovascular or respiratory disease; (2) Combined with Parkinson’s disease, spinal cord injury, multiple sclerosis and other diseases that may affect spasticity or sensory function; (3) Those who are cognitively impaired or unconscious and unable to cooperate with treatment; (4) Joint deformity, fracture, severe arthritis or skin breakage of the lower limb on the affected side; (5) History of gastrocnemius muscle trauma, Achilles tendon contracture, or undergoing lower limb surgery.

This study was reviewed and approved by the Ethics Committee of the Affiliated Hospital of Shandong University of Traditional Chinese Medicine, approval number: (2024) Lun Shen No. (122)-KY; It is also registered on the International Traditional Medicine Clinical Trial Registry Platform, registration number: ITMCTR2025000017.

### 2.2 Evaluation criteria

#### 2.2.1 Assessment of spasticity

The Modified Ashworth Spasticity Scale (MAS) was used to quantitatively evaluate the triceps muscle tone of the calf. The scale was divided into 6 grades: grade 0, grade I., grade I., grade II., grade III., and grade IV., and the patients were divided into mild (grade 1–1) and moderate to severe (≥2) according to the degree of spasticity. The grades were quantitatively scored as 0, 1, 2, 3, 4 and 5, with higher scores indicating more significant muscle tone.

#### 2.2.2 Musculoskeletal ultrasound diagnostic system

**Figure 1:**
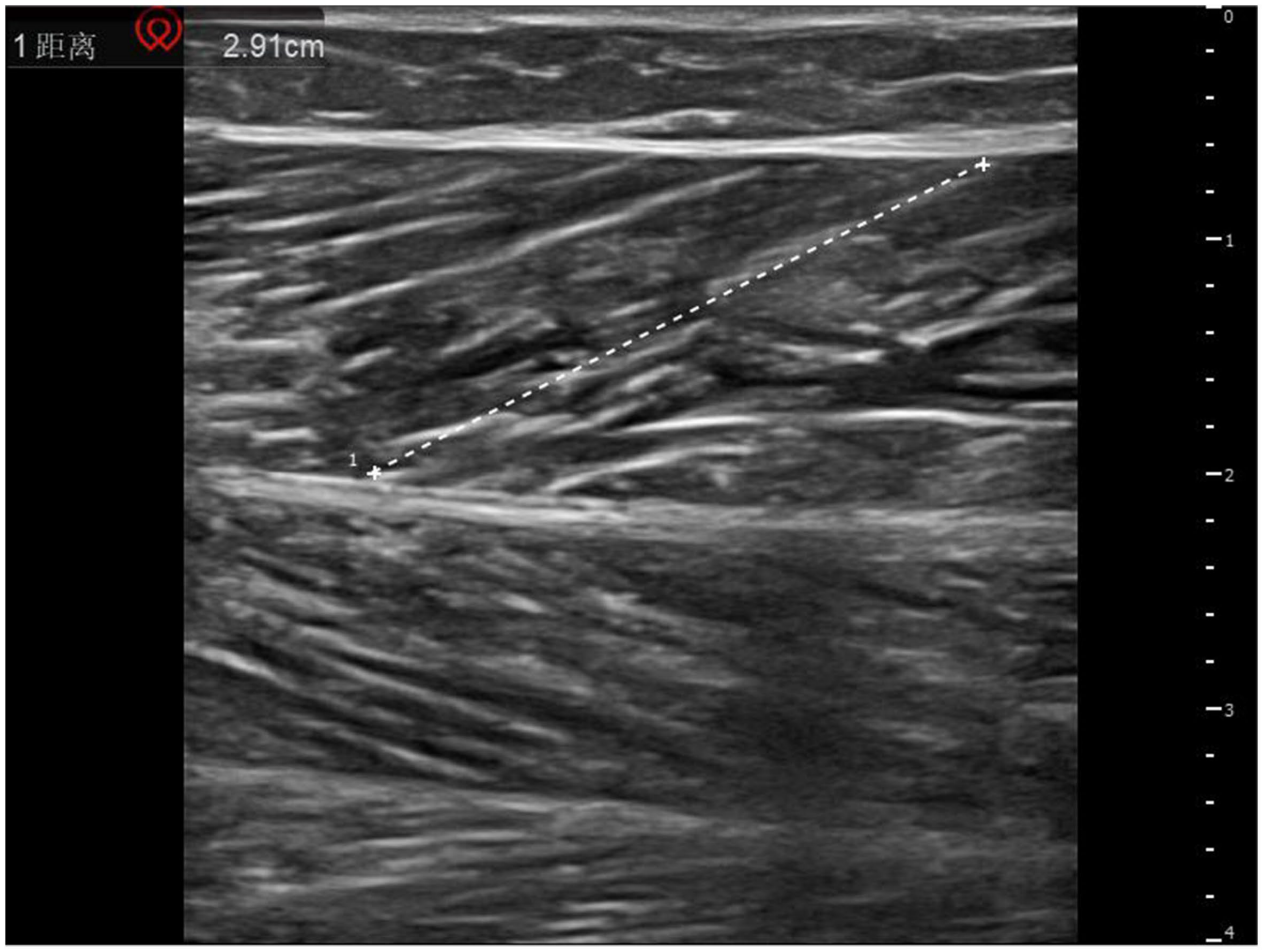
Measurement of myofiber length.

**Figure 2:**
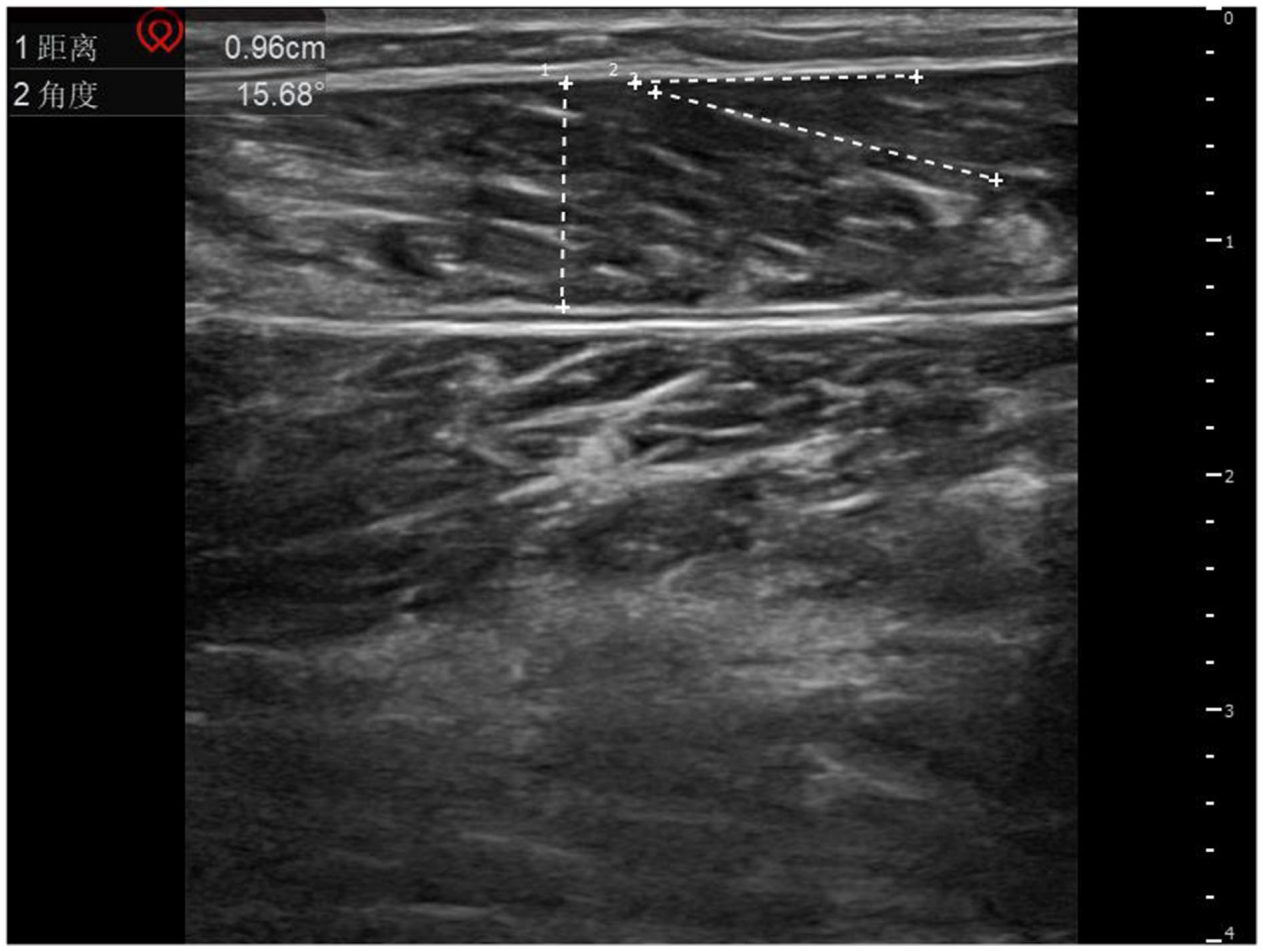
Schematic diagram of muscle thickness and muscle plume angle measurements. 1 represents muscle thickness; 2 represents the muscle plume angle.

A musculoskeletal ultrasound diagnostic instrument (Shenzhen Huasheng Wisonic, model Clover 60) was selected, and the ultrasound probe was selected with a high-frequency probe with a set frequency of 8–12 MHz, and the patient was instructed to lie in a prone position, keep the trunk and lower limbs in a natural relaxed state, and extend both lower limbs and hang the feet on the outside of the bed edge, so that the area below the knee joint on the affected side was fully exposed. The high-frequency linear probe was kept perpendicular to the longitudinal axis of the calf, and the positioning point was selected from the central abdominal area of the medial gastrocnemius cephalic muscle (i.e., the midpoint between 3 cm distal to the skin fold of the popliteal fossa and 5 cm proximal to the Achilles tendon transition area, which is the most abundant area of muscle tissue), and the transverse and longitudinal directions were examined. All ultrasound parameters were measured in a standardized scheme: the length of the muscle fibers (the distance from the muscle-tendon junction to the end of the adjacent muscle bundle in cm) was measured in the longitudinal section, the muscle thickness (the vertical distance from the epimysium to the deep fascia) was measured in the cross-section, and the muscle pinnate angle (the angle between the muscle fibers and the deep fascia in °) was measured in the muscle resting state. See Figures 1, 2 for details.

#### 2.2.3 Proprioceptive assessment

The Pro-kin balance system (PK254 produced by TecnoBody) was used to evaluate the proprioception of the spasticity side of the lower limb, and the multiaxial proprioception assessment plate was used to evaluate it. The patient is instructed to be barefoot, stand on the balance board with the affected foot, naturally hold the armrests on both sides with both hands, look at the front display screen with both eyes, and complete the preset circular trajectory movement of the system through the sole of the foot to control the balance board, and accurately complete the 5-week circular movement within the specified time limit (T ≤ 120 s), and the sound and light alarm will be triggered when the overrun is overrun, and the duration of a single overrun is Δt ≤ 5 s. The synchronous collection indicators include Average Trace Error (ATE) and average weight-bearing asymmetry (AWA). The lower the value, the better the proprioceptive.

### 2.3 Statistical analysis

SPSS 26.0 software was used for data processing. Firstly, the normality test was carried out, and the ^−^x ± s was used for descriptive statistics for the normally distributed quantitative data, in which the paired samples t-test was used for the intra-group difference analysis, and the independent samples t-test was used for the inter-group difference analysis. For the non-normally distributed data, the statistical description was in the form of M (P25, P75), and the Wilcoxon sign rank sum test was used for intra-group comparison, and the Mann–Whitney U rank-sum test was used for comparison between groups. Correlation analysis: Pearson or Spearman correlation analysis was selected according to the characteristics of data distribution. The two-sided test was used, and the test level was *α* = 0.05, and if *p* < 0.05, the difference was statistically significant.

## 3 Results

### 3.1 General information

A total of 80 patients were included in this study, including 38 males and 42 females. Age 25 ~ 80 years old, average (60.10 ± 12.34) years; Height 148 ~ 184 cm, average (165.75 ± 8.32) cm; The BMI was 18.91 ~ 27.44 kg/m2, with an average of (23.72 ± 1.93) kg/m2. The duration of the disease was 36 ~ 176 days, with an average of 104.51 ± 35.75 days. In terms of disease types, there were 36 cases of cerebral infarction (45%) and 44 cases (55%) of cerebral hemorrhage.

### 3.2 MAS score comparison

The degree of spasticity of the lower extremities in stroke is scored as 2 (1, 3). Among them, there were 56 patients with mild spasm, with scores of 1 and 2, and 24 patients with moderate to severe spasm, with scores of 3 and 4.

### 3.3 Musculoskeletal ultrasound assessment results

Compared with the unaffected side, the muscle fiber length, medial head pinnate angle and muscle thickness of the gastrocnemius muscle on the affected side were smaller than those on the healthy side in all spasticity patients (*p* < 0.05) (Table 1).

**Table 1:** Musculoskeletal ultrasound evaluation results, ^−^x ± s.

Constituencies	Number of examples	Muscle fiber length (cm)	Muscle pinnate angle (°)	Muscle thickness (cm)
Affected side	80	2.64 ± 0.50	17.32 ± 3.08	1.20 ± 0.22
Healthy side	80	2.81 ± 0.54	18.92 ± 2.93	1.27 ± 0.23
*t*		−2.003	−3.373	−2.101
*P*		<0.05	<0.05	<0.05

### 3.4 Results of proprioceptive assessment

The mean trajectory difference of all patients was 65.83 ± 13.11 (%), and the mean strength difference was 2.41 ± 0.46 (kg).

### 3.5 Correlation analysis

There was a significant significant difference in MAS score with mean trajectory difference, mean strength difference and muscle pinnate angle, and negatively correlated with muscle fiber length and muscle thickness (*P*<0.05) (Table 2).

**Table 2:** Correlation analysis.

Index	*r*-value				
	ATE	AWA	Muscle fiber length	Muscle pinnate horns	Muscle thickness
MAS score	0.831*	0.808*	−0.455*	0.555*	−0.416*

*Indicates that the correlation is significant and the difference is statistically significant (*P* < 0.05).

### 3.6 Multiple linear regression analysis

MAS score was used as the dependent variable, proprioceptive parameters and muscle structure parameters as independent variables, and a multiple linear regression model was constructed. Multiple linear regression analysis showed that the model had a significant prediction effect on MAS score (*F* = 59.534, *p* < 0.05), and the adjusted R2 was 0.787, indicating that proprioceptive parameters and muscle structure parameters jointly explained 78.7% of the variation in MAS score. The results of this study suggest that proprioceptive function and muscle structure characteristics may be the key factors affecting MAS score (Table 3).

**Table 3:** Multiple linear regression model results.

Argument	*B*	SE	*β*	*t*	*P*
constant	−0.345	0.548		−0.629	0.531
ATE	0.020	0.008	0.329	2.643	0.010
AWA	0.455	0.219	0.260	2.078	0.041
Muscle fiber length	−0.251	0.095	−0.157	−2.654	0.010
Muscle pinnate horns	0.094	0.020	0.362	4.684	<0.001
Muscle thickness	−0.820	0.252	−0.230	−3.251	0.002

### 3.7 Predictive performance of musculoskeletal ultrasound measurement parameters on spasticity severity

**Figure 3:**
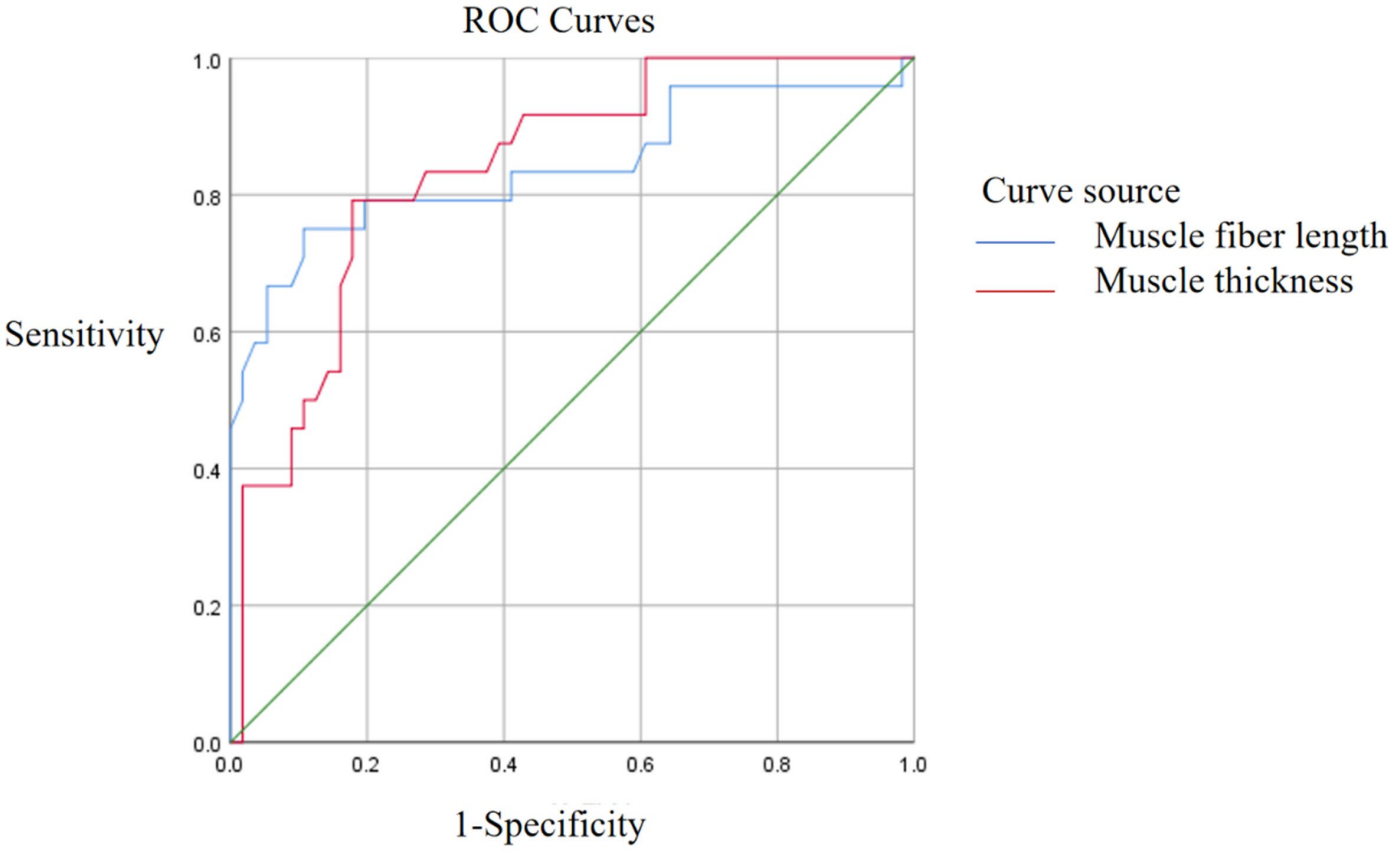
ROC analysis of muscle fiber length and muscle thickness.

**Figure 4:**
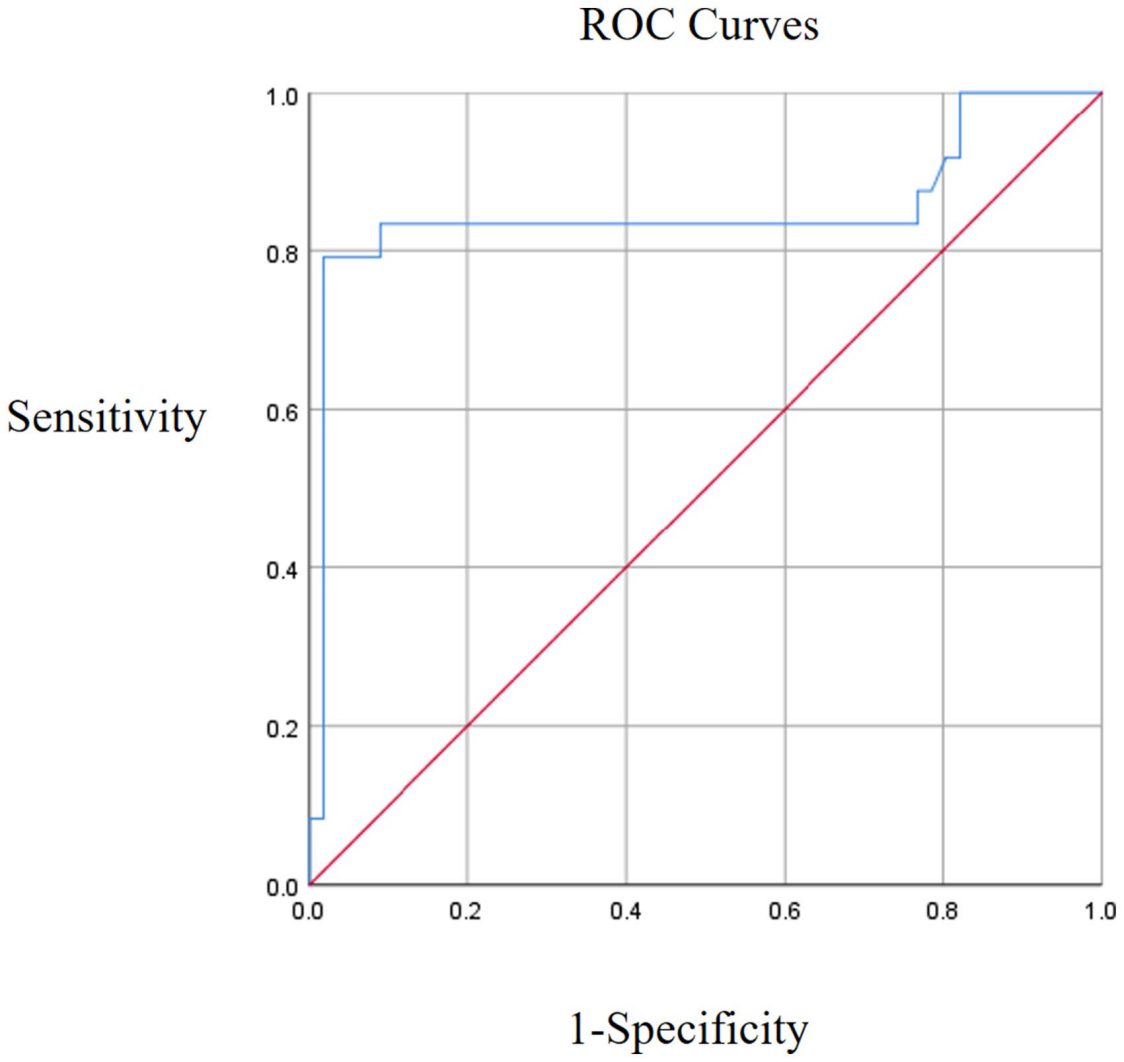
ROC analysis of muscle pinnate angle.

The predictive performance of ultrasound measurement parameters of spasmodic muscle on spasticity degree showed that the predictive performance of spasmodic muscle was the best (AUC = 0.850, 95%CI: 0.729–0.972), and the sensitivity and specificity corresponding to the cut-off value of 19.35 were 79.2 and 98.2%, respectively. The sensitivity and specificity of muscle fiber length (AUC = 0.840, 95%CI: 0.727–0.953) were 75.0 and 89.3%, respectively. Muscle thickness (AUC = 0.838, 95%CI: 0.747–0.929), and the sensitivity and specificity of 1.105 were 79.2 and 82.1%, respectively (Figures 3, 4).

## 4 Discussion

In this study, the musculoskeletal ultrasound system was used to measure the muscle structure parameters of the medial gastrocnemius muscle of the affected lower limb, and the mean trajectory error (ATE) and mean weight-bearing force difference (AWA) were quantitatively detected by the proprioceptive assessment module of the Pro-kin balance system, so as to reveal the relationship between the spasticity and the spasticity, in order to conduct a more comprehensive and systematic study on spasticity, proprioception and muscle structure, and provide a new reference for the rehabilitation evaluation and treatment of lower limb in patients with stroke spasticity.

### 4.1 Medial head structure parameters of gastrocnemius muscle in patients with spasticity

In this study, the musculoskeletal ultrasound diagnostic system was used to evaluate the medial head of the gastrocnemius muscle on the affected side of patients with lower limb spasticity after stroke, and it was found that it showed characteristic morphological changes: compared with the healthy side, the length of the fibers of the medial gastrocnemius muscle on the affected side was shortened, the pinnate angle was reduced, and the muscle thickness was thinned. These changes are consistent with the pathophysiology of muscle fibers in patients with lower limb spasticity, and may have a bidirectional relationship with muscle fiber arrangement disorder and atrophy in spasticity (12). The results show that the length of myofibers is significantly correlated with the dynamic characteristics of contraction, and this parameter directly affects the mechanical transfer efficiency of myofibers along the longitudinal axis during active contraction and passive stretching (13). As a key biomechanical parameter, the muscle pinnate angle is defined as the angle between the direction of the muscle fiber and the longitudinal axis of the tendon. Studies have shown that (14), The shortening of muscle fibers and the reduction of pinnate angle together reduce the mechanical efficiency of muscle contraction, and this biomechanical change can trigger the enhancement of the excitatory reflex of motor neurons *α* in the central nervous system, forming a negative cycle of structural and functional interaction. Muscle thickness is a reflection of the torque of muscle fibers in the direction of the perpendicular perimysium, which reflects the hardness of the muscle sidewall (15). Spasticity of the lower limbs in stroke patients is prone to a synergistic effect with long-term immobilization, which leads to abnormal increase in muscle tone and accelerated protein degradation and metabolism due to limited activity, which together promote the increase of muscle stiffness and the decrease of thickness (16).

### 4.2 Proprioceptive measurement parameters in patients with spasticity

Research shows (17), Lower extremity spasticity is not only involved in motor system injury, but is also closely related to sensory system dysfunction. As the core component of deep sensory, proprioception plays a key role in motion control by transmitting biomechanical information such as limb position, movement speed, and force load in real time through proprioceptors in muscles, tendons, and joints. In this study, the proprioceptive assessment module of the Pro-kin balance system was innovatively adopted, and the objectivity and accuracy of proprioception assessment were significantly improved by quantifying the two-parameter system of ATE (mean trajectory error) and AWA (mean weight-bearing force difference). The average trajectory error can objectively reflect the accuracy of neuromuscular regulation of spatial position during movement by calculating the root mean square error of subjects deviating from the preset trajectory. The symmetry of the mean weight-bearing force difference was quantified by comparing the dynamic data of the bilateral limb pressure sensors, and its sensitivity was significantly improved compared with the traditional one-legged standing test. In this study, it was found that the Pro-kin balance system can accurately assess patients’ proprioception and capture subtle abnormalities of sensory-motor integration more sensitively, and the study of Zhang Xihui pointed out that the system is of great significance for guiding patients’ proprioception training (18).

### 4.3 Relationship between spasticity and proprioception, muscle structure parameters

Correlation analysis showed that the degree of lower limb spasticity in stroke patients was significantly positively correlated with the mean trajectory difference and the average weight-bearing strength difference, and this quantitative study showed that there was a close relationship between the reduced ability of lower limb movement trajectory control and abnormal weight-bearing force regulation in patients with spasticity, and the spasticity caused by central nervous system injury. From the perspective of neural mechanisms, corticospinal tract injury after stroke may affect the sensorimotor integration circuit, resulting in a compensatory imbalance between proprioceptive afferents and motor output (19). Research shows (20), *γ* motor neurons dynamically regulate the sensitivity of the spindle to stretch stimuli by regulating the contraction-diastolic rhythm of the fibers in the muscle spindle, and then participate in the regulation of muscle tone, while the damage to the descending central inhibitory pathway after stroke may lead to abnormal amplification of the muscle spindle afferent signal (21). At the same time, proprioceptive impairment may break the sensory-motor closed-loop regulation at the spinal cord level, making *α* motor neurons overexcited, and then inducing and maintaining spasticity, and finally forming a vicious circle of “increased muscle tone-abnormal motor pattern-distortion of proprioceptive input.” At the level of muscle structure, this study found that there was a significant negative correlation between muscle fiber length, muscle thickness and MAS score, and the mechanism may be related to the decrease in the number of myoscle tandems and myofibrillary remodeling caused by persistent spasticity. Shortening of the abdomen may decrease muscle compliance and increase resistance in passive stretch (22). Persistent hypertonia causes myofibers to remain in a non-functional shortening state for a long time, accelerates the degradation of myofibrillar proteins, and leads to a reduction in the cross-sectional area of myofibers. Consistent with the findings of Serdar (23), the pinnate angle of muscles was positively correlated with MAS score, and an increase in the pinnate angle may reflect the disordered arrangement of muscle fibers and the increased degree of fibrosis, suggesting an increase in muscle stiffness.

Multiple linear regression analysis further suggested that proprioceptive function and muscle structure characteristics may be the key factors affecting spasticity scores, suggesting that muscle morphological changes may affect spasticity phenotype by altering biomechanical properties and sensory dysfunction. In this study, muscle structure parameters (such as pinnate angle, muscle fiber length, etc.) were included in the regression model for the first time, and it was found that they were independently correlated with MAS scores, and it was speculated that muscle structure remodeling may affect muscle stiffness and stretch reflex threshold by changing the arrangement of muscle tracts and contraction efficiency. This is consistent with the study of Xia (24), jointly elucidate the biomechanical basis of muscle morphological parameters in spasticity. At the same time, this study confirmed that proprioception has important clinical value in the spasticity rehabilitation of stroke patients, which is manifested in the fact that the proprioceptive system regulates the excitability of *α* motor neurons through the *γ*-muscle spindle afferent pathway, and its dysfunction may lead to weakened central inhibition and hyperreflexia, this is consistent with the study by Zheng (25).

In conclusion, post-stroke spasticity involves the interaction of central regulatory abnormalities (dysregulation of sensorimotor integration and diminished downward inhibition) and peripheral adaptive changes (changes in muscle structure characteristics such as muscle fiber structural remodeling), which provides a theoretical basis for spasticity-targeted interventions (such as rehabilitation strategies combining anticonvulsive drugs, proprioceptive training, and adjustment of muscle mechanical properties).

### 4.4 Ultrasound measures the predictive analysis of muscle structure parameters for different degrees of spasticity

In this study, the prediction model of mild and moderate to severe spasticity subgroups was established, and the differences in the predictive performance of muscle pinnate angle, muscle fiber length and muscle thickness on spasticity severity were revealed, suggesting that musculoskeletal ultrasound has good diagnostic efficacy in measuring spasticity muscle structure parameters. ROC curve analysis showed that the musculoskeletal ultrasound parameters in the three groups showed good diagnostic value, among which the pinnate angle had the best predictive performance, and its high specificity suggested that this parameter had a significant advantage in distinguishing patients with mild and moderate to severe spasticity. From the perspective of physiological mechanism, the pinnate angle reflects the arrangement angle of muscle fibers and tendons, and the increase of muscle tone in the spasticity state may lead to the remodeling of muscle fibers, which is manifested as the increase or decrease of the pinnate angle, which is closely related to the three-dimensional spatial conformation of muscle spindle and surrounding muscle fibers (26). Research shows (27), In the spastic state *γ* excessive excitation of motor neurons leads to increased sensitivity of muscle spindle, and the geometric characteristics of pinnate angle can intuitively characterize the mechanical coupling efficiency of muscle spindle receptors. Compared with traditional parameters such as myofiber length or cross-sectional area, the three-dimensional properties of the pinnate angle allow it to better capture the nonlinear changes in the microstructure during dynamic muscle contraction, which may be the reason why its prediction performance is significantly better than that of traditional parameters. The predictive power of muscle fiber length and muscle thickness is similar, but there are differences in clinical significance. In contrast, the sensitivity and specificity of myofiber length suggest that it is more suitable for screening for moderate to severe spasticity, and its cut-off value may be related to muscle fiber shortening due to spasticity (28). The sensitivity and specificity of muscle thickness are relatively balanced, and the mechanism of change may be related to compensatory hypertrophy caused by continuous contraction of spasmodic muscle groups. The combination of the two may be complementary: the length of the muscle fibers focuses on structural changes, and the muscle thickness reflects the immediate state of tension. Future research will explore multi-parameter joint models to further improve prediction accuracy.

Therefore, based on the results of this study, musculoskeletal ultrasound parameters and proprioceptive indexes can provide guidance for the clinical management of lower limb spasticity in stroke. When the medial head pinnate angle of the gastrocnemius muscle is > 19.35°, the muscle fiber length < 2.31 cm, or the muscle thickness < 1.105 cm, the risk of moderate to severe spasticity is suggested. For botulinum toxin injection, ultrasound-guided targeted injection into the pinnate angle enlargement area is used to enhance the efficacy. In the future, ultrasound parameters and spasticity scores can be combined to regularly evaluate the changes in spasticity degree and achieve precise rehabilitation.

There are also some limitations in this study, firstly, the sample size is small, and the sample size will be expanded in combination with multiple centers. Secondly, the cross-sectional study design has not yet established a definite causal relationship, and a longitudinal study can be designed in the future to systematically evaluate the long-term efficacy of proprioceptive training on spasticity improvement in a randomized controlled trial, and the intervention mechanism will be analyzed in combination with multimodal neuroimaging technology.

## 5 Conclusion

There is a significant correlation between lower limb spasticity and proprioception and muscle structure parameters in stroke patients, proprioception and muscle structure parameters are the key factors affecting spasticity, and musculoskeletal ultrasound measurement parameters can have good predictive value for spasticity degree, which can provide an objective quantitative basis for clinical evaluation.

## Data Availability

The raw data supporting the conclusions of this article will be made available by the authors, without undue reservation.
